# Babesias of red deer (*Cervus elaphus*) in Ireland

**DOI:** 10.1186/1297-9716-42-7

**Published:** 2011-01-18

**Authors:** Annetta Zintl, Eugene J Finnerty, Thomas M Murphy, Theo de Waal, Jeremy S Gray

**Affiliations:** 1 UCD School of Agriculture, Food Science and Veterinary Medicine, University College Dublin, Ireland; 2 UCD School of Biology and Environmental Science, University College Dublin, Ireland; 3 Central Veterinary Laboratories, Backweston, Co. Kildare, Ireland

## Abstract

Blood samples were obtained from 38 wild red deer (*Cervus elaphus*) at two sites in Ireland and subjected to PCR analysis of the 18S rRNA gene followed by sequencing. Two fragments of the 18S rRNA gene were generated by two different PCR protocols and subsequent sequencing suggested that at least six of the deer were infected by a babesia that, in those loci, is indistinguishable from *Babesia divergens*, an important tick-borne pathogen of cattle and of zoonotic significance. Additionally, a *B. odocoilei*-like parasite was detected in three samples and a babesia that did not match any sequences in the GenBank database was found in five samples. Neither *B. capreoli *nor *B. venatorum *(EU1) were found. There have been several reports of *B. divergens *occurring in deer species, including red deer, roe deer (*Capreolus capreolus*) and reindeer (*Rangifer tarandus*). However, in view of recent re-sequencing of bovine-origin samples deposited previously in GenBank, it is unlikely that any of these sequences from deer are *B. divergens*. The present study describes the only deer piroplasm detected so far that shows complete identity with *B. divergens*, in just over half of the 18S rRNA gene. The entire gene of this deer parasite should be analysed and transmission experiments undertaken before the infectivity of *B. divergens *for red deer can be confirmed.

## Introduction

There has been considerable recent interest in the identity of *Babesia *spp. in deer, because of concerns about the health of endangered host species such as chamois (*Rupicapra r. rupicapra*) [1] and also because of the possibility of deer acting as reservoirs for important cattle parasites such as *Babesia divergens *[2]. Earlier studies on red (*Cervus elaphus*) and sika (*C. nippon*) deer babesias in Europe suggested that they were morphologically and antigenically indistinguishable from *B. divergens*, but were not transmissible to splenectomised calves. They were therefore tentatively identified as *B. capreoli *[3,4], which was first observed in roe deer (*Capreolus capreolus*) [5]. With the advent of molecular taxonomy based on analysis of DNA sequences, several authors described parasites in roe, red or reindeer as *B. divergens *or *B. divergens-*like [2,6-12]. These authors based their conclusions on 18S rRNA gene sequence alignment, but none of their samples showed 100% similarity with *B. divergens *sequences of bovine origin in GenBank. Resequencing of the 18S rRNA gene from the same strains of *B. divergens *originally deposited in GenBank showed that all were in fact identical and that there were errors in the original sequences (Slemenda et al., unpublished, cited in [13]). This suggests that parasites showing less than 100% similarity for this gene should not be designated *B. divergens *and to date there are no studies showing that *B. divergens *occurs naturally in ruminant hosts other than cattle.

An opportunity to re-examine this situation arose during a serosurvey of deer parasites in free-ranging wild Irish deer. Blood samples from 38 red deer from two National Parks in different geographical locations were analysed by two PCR protocols targeting the 18S rRNA gene, and the products then sequenced and aligned for identification purposes.

## Materials and methods

**Figure 1:**
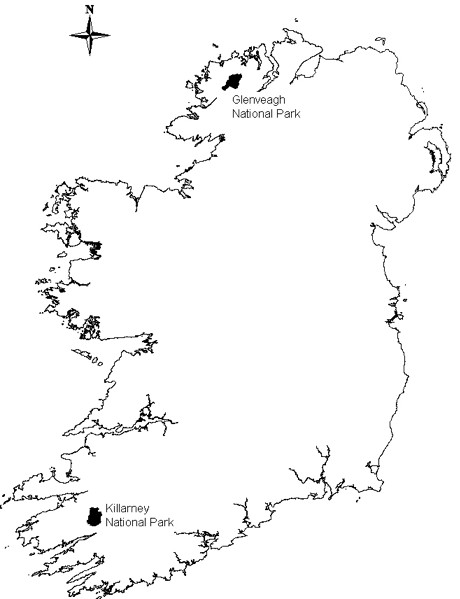
**Locations of sites where deer were sampled**.

Blood samples were collected from red deer shot by the National Parks and Wildlife Service as part of the seasonal cull in Glenveagh (n = 27) and Killarney (n = 11) (Figure 1). From each animal 3 to 4 mL whole blood were collected into EDTA. Following centrifugation and removal of plasma, the packed cell component was stored at -20°C. DNA was subsequently extracted from thawed and mixed 100 mg packed cells of each sample using the High Pure PCR Template Preparation Kit (Roche, Burgess Hill, UK). A nested PCR protocol was used initially to screen all samples (protocol I). The positive samples were then additionally analysed using a hemi-nested PCR protocol (protocol II). The two PCR protocols target different regions of the 18S rRNA gene and are modifications of previous published assays. Details are provided in Table 1. *B. divergens *DNA extracted from a bovine isolate was used as a positive control. Negative controls were performed in the absence of template DNA. PCR products were fractionated on 2% agarose gels and visualised by staining with SYBR Safe DNA gel stain (Invitrogen, Paisley, UK). Amplicons were purified using the QIAquick PCR purification kit (Qiagen, Crawley, UK) and sequenced (GATC Biotech AG, Konstanz, Germany). Consensus sequences were obtained from between 5 to 14 forward and 2 reverse sequences, each. Comparisons were made with published sequences using NCBI Blast, aligned with the ClustalW2 sequence alignment programme and logged in GenBank under accession numbers GU475472 to GU475475. Because of insufficient sequence overlap at the 5'end, logged sequences are approximately 40 bp shorter than the amplicons.

**Table 1:** Details of the nested and hemi-nested PCR protocols used to screen deer blood samples for the presence of *Babesia *spp.

PCR protocol	Primers (positions)*		Amplification protocol	Reference
I	1^st ^PCR:			
Product size*:	BTH-1F (365-385)	5'cct gag aaa cgg cta cca cat ct	94°C: 10 min,	[23]
561 bp	BTH-1R (1031-1050)	5' ttg cga cca tac tcc ccc ca	40 cycles: 95°C: 30 s, 68°C: 1 min, 72°C: 1 min	
			72°C: 10 min	
	nested PCR:			
	GF2 (466-487)	5' gtc ttg taa ttg gaa tga tgg	94°C: 10 min	[19]
	GR2 (1006-1026)	5' cca aag act ttg att tct ctc	40 cycles: 95°C: 30 s, 60°C: 1 min, 72°C: 1 min	
			72°C: 10 min	
II	1^st ^PCR:			
Product size*:	Babfor (959-977)	5'gac tag gga ttg gag gtc	94°C: 10 min	[24]
576 bp	Babrev (1589-1610)	5'gaa taa ttc acc gga tca ctc	35 cycles: 95°C: 1 min, 53°C: 1.5 min, 72°C: 1.5 min	
			72°C: 10 min	
	nested PCR			
	BT2-F (1035-1055)	5'gga gta tgg tcg caa gtc tg	94°C: 10 min	[23]
	Babrev		35 cycles: 95°C: 1 min, 53°C: 1.5 min, 72°C: 1.5 min	
			72°C: 10 min	

* product sizes and primer positions according to reference sequence AY046576

Phylogenetic analysis of the relationship between sequences of our isolates and published sequence data was carried out using MEGA version 3.1 [14]. This software programme was used to construct a Neighbour-Joining tree. Tree reliability was assessed by the bootstrap method with 1000 pseudoreplicates.

## Results

**Figure 2:**
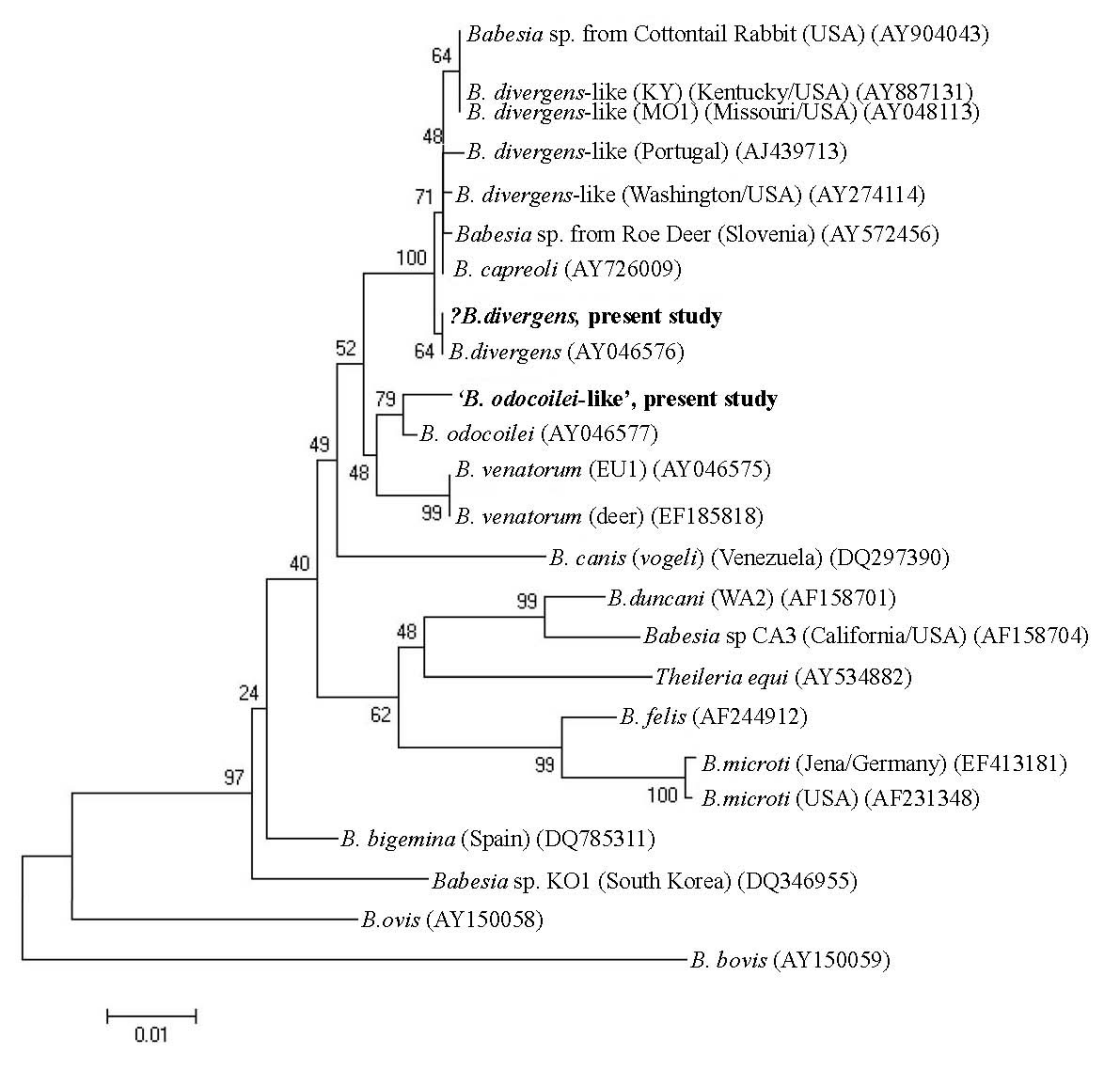
**Phylogenetic relationships of deer *Babesia *species (present study in bold) according to the sequence amplified by PCR protocol I (bp loci 527-1005 in reference sequence **AY046576*). The tree was constructed by sequence alignment of the 18S rRNA gene fragment and neighbour-joining analysis. Tree reliability was assessed by the bootstrap method with 1 000 pseudoreplicates (MEGA version 3.1, [14]). (*all amplicons exclude the primer sequences (as they are not part of the original DNA template) and approx. 40 bp at the 5' region for which there was insufficient over-lapping sequencing data).

**Figure 3:**
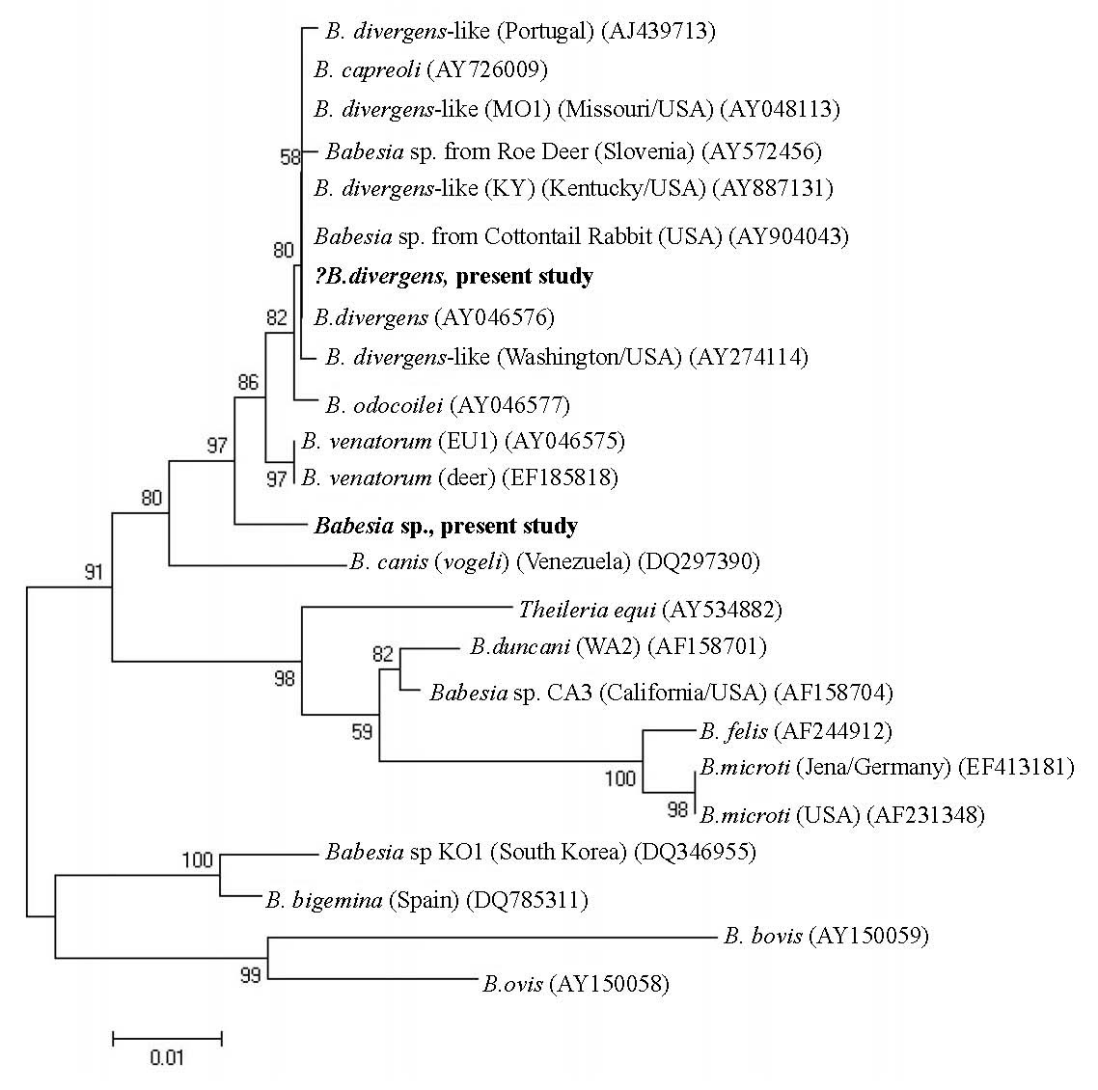
**Phylogenetic relationships of deer *Babesia *species (present study in bold) according to the sequence amplified by PCR protocol II (bp loci 1096-1589 in reference sequence **AY046576*). The tree was constructed as described for Figure 2.

The initial screen using PCR protocol I revealed that 18 deer carried *Babesia *spp. infections (26%), with 17 originating from Glenveagh and 1 from Killarney (Table 2). The *Babesia *species present in these animals were identified by sequence-analysis of fragments amplified using both PCR protocols. Six samples from Glenveagh showed 100% similarity to *B. divergens *(GenBank AY046576) in both fragments of the 18S rRNA gene (logged in GenBank under accession numbers GU475472 (PCR protocol I) and GU475473 (protocol II)). In a further five isolates one amplicon (resulting either from PCR protocol I or II) showed 100% *B. divergens *similarity, although the second amplicon could only be identified to genus level. In addition, identical nested PCR products (amplified using protocol I) from three Glenveagh deer samples were 98% similar to *B. odocoilei *(GenBank AY046577) and have been logged as GU475474. Five products from PCR protocol II (4 from Glenveagh and 1 from Killarney) were also identical to each other but did not closely match any *Babesia *species in the GenBank database (96% similarity with *B. divergens*, *B. odocoilei *and EU1) (accession number GU475475) (Table 2). Figure 2 shows the phylogenetic positions of the amplicons arising from PCR protocol I, which proved to be more discriminating than PCR protocol II. The latter protocol generated amplicons that did not differentiate bovine-origin *B. divergens *from any of the *B. divergens*-like species, but showed that the unknown *Babesia *sp. is clearly separate from *B. divergens, B. odocoilei *and *B. venatorum *(Figure 3). The remaining positive PCR amplicons could only be identified to *Babesia *genus level due to poor sequencing data.

**Table 2:** Presence of *Babesia *spp. in red deer whole blood samples from Glenveagh and Killarney.

Glenveagh			
Deer N°	+/-	species ID according to product I	species ID according to product II

G1	+	98% identical with B. odocoilei*	96% identical with *B. odocoilei*, *B. divergens*, EU1**

G2	+	100% identical to B. divergens***	100% identical to B. divergens***

G3	+	98% identical with B. odocoilei*	96% identical with *B. odocoilei*, *B. divergens*, EU1**

G4	-		

G5	+	100% identical to B. divergens***	*Babesia *sp****

G6	+	100% identical to B. divergens***	100% identical to B. divergens***

G7	+	*Babesia *sp****	96% identical with *B. odocoilei*, *B. divergens*, EU1**

G8	-		

G9	-		

G10	+	*Babesia *sp****	96% identical with *B. odocoilei*, *B. divergens*, EU1**

G11	-		

G12	-		

G13	+	100% identical to B. divergens***	100% identical to B. divergens***

G14	+	100% identical to B. divergens***	100% identical to B. divergens***

G15	+	*Babesia *sp****	100% identical to B. divergens***

G16	+	*Babesia *sp****	-

G17	-		

G18	+	100% identical to B. divergens***	100% identical to B. divergens***

G19	+	100% identical to B. divergens***	-

G20	-		

G21	-		

G22	+	*Babesia *sp****	Babesia sp****

G23	+	100% identical to B. divergens***	100% identical to B. divergens***

G24	-		

G25	+	100% identical to B. divergens***	Babesia sp****

G26	-		

G27	+	98% identical with B. odocoilei*	Babesia sp****

Killarney			

Deer N°	+/-	species ID according to product I	*species ID according to product II*

KR1	-		

KR2	-		

KR3	-		

KR4	-		

KR5	+	100% identical to B. divergens***	96% identical with B. odocoilei, B. divergens, EU1**

KR6	-		

KR7	-		

KR8	-		

KR9	-		

KR10	-		

KR11	-		

*Babesia *spp were identified by sequence allignment of amplification products from PCR protocols I and II (products I and II respectively).

* sequence identical between G1, G3 and G27 (reference sequence: AY046577)

** sequence identical between G1, G3, G7, G10, KR5 (reference sequences: AY046575, AY046576, AY046577)

*** reference sequence: AY046576

**** amplicons only identifiable to genus level due to poor sequencing data

## Discussion

Reports of the identification of *B. divergens *in reindeer [8], roe deer [2,6,9,12], red deer [2] and chamois [1] were noteworthy because this parasite is not only an important pathogen of cattle but is also zoonotic, causing a dangerous fulminating disease in splenectomised patients [15,16]. The presence of *B. divergens *in free-ranging wild deer would affect our understanding of both bovine and human babesiosis epidemiology. However, reliance on similarity of fragments of the 18S rRNA gene for identification of this parasite is questionable. Although this gene is of great taxonomic value within the Apicomplexa, it has been suggested that it may not discriminate well between very closely related parasites [11]. Furthermore, few of the recent studies on babesia in European deer analysed the whole gene, or attempted infection with deer parasites of splenectomised cattle or gerbils (*Meriones unguiculatus*), both of which infections are reliable markers for *B. divergens *identity [16]. The most complete sequences analysed were from babesias in farmed reindeer in Scotland [8] and from roe deer in Slovenia [2]. In both these studies it was concluded that the high level of similarity (99.8 and 99.6% respectively) when compared with *B. divergens *GenBank depositions made in the mid-90s (U16370, UO7885 and Z48751) justified identity with *B. divergens. *However, when the whole 18S rRNA gene from the original *B. divergens *strains were resequenced, along with several others, it was found that all were identical and the only strain that had been accurately sequenced among the early GenBank depositions was U16370 (Slemenda et al., unpublished, cited in [13]). This calls into question *B. divergens *identity of any isolates showing less than 100% similarity to U16370. Although further bovine *B. divergens *sequence data were recently deposited in GenBank, these show only one (EF458223, EF458224, EF458227) or two (EF458219) base pair differences from the approximately 12 accurate sequences of other *B. divergens *bovine isolates in the data base. None of the deer-derived babesia isolates referred to above showed less than four base-pair differences and in only one study [8] was an attempt made to infect gerbils. No infection occurred but this could have been due to reduced viability of the parasites resulting from the delay in their arrival at the laboratory by mail.

The tentative identification of *B. divergens *in the present study is based on analysis of two fragments of the 18S rRNA gene (bp loci 527-1005 and 1096-1589 in the bovine origin reference sequence, AY046576), more than half the total gene of 1728 bp. In the other publications that reported detection of *B. divergens *and where a significantly large proportion of the gene was analysed, base pair differences compared with AY046576 occurred at the following base pair loci - 632, 663, 1277 (AY098643[8]); 373, 631, 663, 804, 1365, 1423 (AY572456[2]), whereas no differences from our sequences were found at these loci. Schmid et al. [11] detected *B. divergens*-like piroplasms with a similar level of 18S rDNA similarity (99.5% and 99.6%) in roe deer and chamois. They did not identify these parasites as *B. divergens *and suggested they should be regarded as *B. capreoli*, although the sequences (EU182596 and EU182597) do not show 100% 18S rDNA similarity with this species either (AY726009). It is possible that bovine *B. divergens*, which evidently shows negligible variability in the 18S rRNA gene [13,17], is a variant of deer origin that has developed specificity for cattle.

In striking contrast to the above three studies, the *B. divergens*-like sequences from the present study showed no differences from bovine *B. divergens *sequences at base pair loci 527-1005 and 1096-1589. Whether or not this parasite is truly identical with bovine *B. divergens *requires further study, but this is also the first reliable demonstration of a *B. divergens-*like piroplasm (even if not *B. divergens*) in red deer because the only previous study that considered this deer species [2], apparently did not attempt to sequence the PCR product.

The highest proportion of babesia-infected deer occurred in the Glenveagh herd and most identifiable babesia sequences were also obtained from this herd. Although serology detected antibodies in the majority of the Killarney samples (unpublished data), no identifiable amplicons could be generated by PCR. It is not obvious why these differences occurred. The deer in all the locations were exposed to heavy tick (*Ixodes ricinus*) challenge judging by the numerous attached ticks observed at culling.

It is of interest that neither *B. capreoli *nor *B. venatorum *were found in the present study. *B. capreoli *was first described by Enigk and Friedhoff [5], in roe deer based primarily on morphology. Adam and Blewett [3], and Gray et al. [4], tentatively identified parasites isolated from red deer and sika deer respectively as *B. capreoli*, based on origin, morphology and antigenicity. In both cases transfer to splenectomised calves failed to result in infection, suggesting that these parasites were not *B. divergens*, though in the light of recent DNA analysis data it seems likely that they were *B. divergens*-like parasites. More recently, DNA analysis by Hoby et al. [7] and Malandrin et al. [18] confirmed that *B. capreoli *occurs in roe deer and the former authors also detected this parasite in chamois and red deer, though single samples only were positive in these latter host species. *B. venatorum *(EU1) first came to scientific notice as a zoonotic infection [13], and has since then been firmly associated with roe deer [18,19], but has not so far been detected in red deer. The apparent absence of both *B. capreoli *and *B. venatorum *in the present study correlates well with the fact that roe deer do not occur in Ireland [20].

*B. odocoilei *is a piroplasm of American white-tailed deer (*Odocoileus virginianus*) and close relatives of this parasite have been detected in European ticks (*I. ricinus*) by PCR [13,21]. Unfortunately no sequence data are available from the PCR products of the tick analyses for comparison with the present study, which appears to be the first record of such a piroplasm in European deer, though the *B. odocoilei*-like parasite detected by Hilpertshauser et al. [22] was in ticks removed from roe deer and which therefore contained deer blood. One further sequence in the present study was identifiable as *Babesia *sp. but did not match any sequence in GenBank. Since it was identical in samples from five individual deer, it may represent a new babesia 18S rDNA sequence.

Analysis of the complete 18S rRNA gene would, of course, have provided a greater level of confidence in the identity of the babesias detected in this study. Unfortunately, available resources did not permit this. Nevertheless, the gene fragment analysis presented here suggests that there are at least two *Babesia *species, if not three, in the red deer sampled in this study. The significance of these parasites as disease agents is unknown and further studies, in addition to gene analysis, including isolation of the parasites and transmission studies in in vitro or in vivo systems are necessary to establish their identities, particularly that of the putative *B. divergens.*

## Competing interests

The authors declare that they have no competing interests.

## Authors' contributions

AZ performed the DNA analysis and was joint lead author of the manuscript, EF and TM collected the samples and participated in writing the manuscript, TDW provided advice on DNA analysis and participated in writing the manuscript, JG planned and co-ordinated the study and was joint lead author of the manuscript. All authors read and approved the final manuscript.
